# Cai’s prescription inhibits granulosa cell apoptosis through *ARHGAP4* on poor ovarian responders

**DOI:** 10.1186/s13048-024-01363-x

**Published:** 2024-02-14

**Authors:** Zheng Wang, Denghao Liu, Yonghong Nie, Qinhua Zhang

**Affiliations:** 1 Department of Integrated Traditional Chinese and Western Medicine, Shanghai First Maternity and Infant Hospital, Tongji University, Shanghai, 200040 China; 2 Prenatal Diagnosis Center, Shanghai First Maternity and Infant Hospital, Tongji University, Shanghai, 200040 China

**Keywords:** Poor ovarian response, Traditional Chinese medicine, *ARHGAP4*, Apoptosis

## Abstract

**Purpose:**

Poor ovarian response (POR) is a big challenge for in vitro fertilization. The traditional Chinese medicine, Cai’s Prescription of Tonifying Kidney and Strengthening Vitals (Cai’s Prescription) has yielded satisfactory results for POR treatment clinically, but systematic scientific research of Cai’s Prescription is not well reported. This study aimed to investigate the clinical effect of Cai’s Prescription on poor ovarian responders and its biological mechanism.

**Methods:**

Serum was collected from poor ovarian responders, and IL-1β, INFγ, FSH, E_2_ and AMH levels were analyzed by ELISA. Ovarian antral follicles were identified and counted using transvaginal ultrasound. The embryo quality grading were done on day 3 after retrieval. We used high-throughput sequencing of granulosa cells to investigate the gene transcription patterns of ovarian granulosa cells in poor ovarian responders after Cai’s Prescription pretreatment. The expression level of *ARHGAP4* was analyzed by quantitative real-time PCR and western blot. The effects of *ARHGAP4* for granulosa cells were analyzed by CCK-8 assay, annexin-V and PI staining, ELISA and western blot. The effects of Cai’s Prescription on the expression of PI3K-Akt pathway and apoptosis were analyzed by western blot.

**Results:**

In this study, we found that Cai’s Prescription pretreatment had the tendency to improve the ovarian reserve function and could increase the number of high quality embryos for poor ovarian responders. Through high-throughput sequencing of mRNA in granulosa cells, we discovered *ARHGAP4*, which is a member of GTPase-activating proteins (GAPs) may be a candidate target for POR treatment. *ARHGAP4* was significantly increased in poor ovarian responders and can be recovered after Cai’s Prescription pretreatment. Mechanically, combining the cell line model and clinical tissue samples, we found that *ARHGAP4* can accelerate cell apoptosis and inflammation response in granulosa cells via PI3K-Akt signaling pathway. In addition, Cai’s Prescription pretreatment for three months significantly reduced the high level of *ARHGAP4* in poor ovarian responders.

**Conclusion:**

This study shows that the traditional Chinese medicine, Cai’s Prescription yielded satisfactory results for poor ovarian responders clinically and *ARHGAP4* may be a candidate target for POR treatment.

**Supplementary Information:**

The online version contains supplementary material available at 10.1186/s13048-024-01363-x.

## Introduction

Despite the considerable progress to assisted reproductive technology (ART) over the last 40 years, the clinical management of patients with poor ovarian response (POR) is still a big challenge in everyday practice, frustrating to both the patient and the fertility expert [1, 2]. POR is characterized by low ovarian response to gonadotropin (Gn) stimulation [3]. Patients with poor ovarian response have fewer antral follicles, low reactivity to exogenous gonadotropins, fewer eggs taken, and fewer embryos available for transplantation, resulting in lower clinical pregnancy rate and poor prognosis, which are very difficult problems in human ART [4, 5].

The suggested globally incidence of POR accounts for about 20% of patients undergoing ovarian stimulation for in vitro fertilization (IVF) [6]. Various controlled ovarian hyperstimulation protocols and strategies have been used in this group of women to improve reproductive outcome, such as the early-follicular-phase long-acting GnRH-agonist long (EFLL) protocol, mid-luteal-phase short-acting GnRH-agonist long (MLSL) protocol and gonadotropin-releasing hormone antagonist (GnRH-ant) protocol, but the success rate still remains very low [7, 8].

The mechanism of POR was complicated and still got no clear conclusion. Suggested etiopathogenesis for POR include: age-related depletion of ovarian follicles, advanced endometriosis, basal hormone levels, anti-mullerian hormone (AMH), chromosomal and genetic alterations, prior ovarian surgery and pelvic adhesions, metabolic and enzymatic diseases, as well as toxic, autoimmune and infectious diseases [9, 10]. At present, the specific mechanism for clinical treatment of ovarian hyporesponsiveness is still under discussion, and effective treatment methods for poor ovarian responders have become major challenges in assisted reproduction [11].

In China, traditional Chinese medicine (TCM) has been employed to cure different kinds of diseases for more than 2000 years [12]. Meanwhile, TCM has been used to help couples who can not get pregnant to conceive in more low-tech ways [13]. Recently, TCM multi-channel interventional therapy on in vitro fertilization-embryo transfer (IVF-ET) failure cases has attracted increasing attention. For example, Antai Recipe can increase the fertility rate, clinical pregnancy rate, and decrease the early abortion rate during the period of secondary IVF-ET for patients with ART Failure [14]. The traditional Chinese formula Ding-Kun Pill (DKP) supplementation in an IVF/ICSI cycle can improve the number of high-quality blastocysts in expected poor ovarian response women [15].

The traditional Chinese medicine Cai’s Prescription of Tonifying Kidney and Strengthening Vitals (Cai’s Prescription) invented by Xiaosun Cai, a traditional Chinese doctor in China, was widely used for poor ovarian responders and yielded satisfactory results in Chinese hospitals for several decades. However, so far, clinical and experimental researches on Cai’s Prescription regarding its regulation and molecular mechanisms in the context of POR are still lacking. In the present study, we found Cai’s Prescription pretreatment had the tendency to improve the ovarian reserve function of poor ovarian responders, and increase the number of high quality embryos. Through high-throughput sequencing of mRNA in granulosa cells from normal and poor ovarian responders, we found that *ARHGAP4*, a member of GTPase-activating proteins (GAPs), mediates apoptosis and inflammation in granulosa cells via PI3K-Akt signaling pathway.

## Materials and methods

### Composition and preparation of Cai’s prescription

Cai’s Prescription of Tonifying Kidney and Strengthening Vitals purchased from Lei Yun Shang Pharmaceutical Group Co. (Suzhou, China) consisted of 11 herbs: Fuling (Indian Bread), 12 g; Shengdihuang (Dried Rehmannia Root), 10 g; Shudihuang (Prepared rhizome of Adhesive Rehmannia ), 10 g; Nvzhenzi (Glossy Privet Fruit), 10 g; Xianmao (Common Curculigo Rhizome), 10 g; Xianlingpi (Epimedium Herb) 10 g; Bajitian (Morinda Root), 10 g; Roucongrong (Cistanche), 10 g; Lujiaoshuang (Degelatined Deer-horn), 10 g; Zishiying (Fluorite), 30 g; and Ziheche (Dried Human Placenta), 3 g. The medicinal materials are processed according to requirements.

### Participants collection

This study included a total of 90 patients received IVF-ET between January 2019 and December 2021. The patients in the normal group (*n* = 30, 38.77 ± 2.47 year old) were infertile woman with normal ovarian function, such as obstruction of fallopian tubes or endometriosis. Poor ovarian responders were diagnosed with the Bologna criteria: (1) advanced maternal age (≥ 40 years), (2) a previously characterized POR cycle (≤ 3 oocytes with a conventional stimulation protocol), (3) an abnormal ovarian reserve test [antral follicle count (AFC) < 5–7 follicles or AMH < 0.5–1.1 ng/mL]. At least two of the above three features must be present for poor ovarian responders [16]. Moreover, all patients with the age < 45 years old have received at least one ovarian hyperstimulation and no other traditional Chinese medicine has been taken in recent three months.

Poor ovarian responders with the following symptoms will be excluded from this study: previous ovarian surgery or pelvic radiotherapy; untreated hydrosalpinx; uterine malformation shape or intrauterine adhesions; serious primary diseases such as cardiovascular, liver, kidney and hematopoietic system; psychotic patients; chromosome abnormality.

A total of 60 poor ovarian responders were randomly allocated into either POR group (*n* = 30, 38.47 ± 4.08 year old) or Treat group (*n* = 30, 38.93 ± 2.46 year old).

### Therapeutic plan and oocyte retrieval

The decoction of Cai’s Prescription is generally prepared by Lei Yun Shang Pharmaceutical Co., LTD. The participants obtained Cai’s Prescription (150 ml) orally twice a day 1 h after breakfast and dinner for three months, except menstrual period. The normal group and POR group did not need to take drugs within three months in the same period. Then, all participants underwent ovarian hyperstimulation according to the GnRH-ant protocol on menstrual cycle day 2 or 3 [17]. Briefly, all participants were administered 225-300IU recombinant FSH (rFSH, Merck Serono, Germany) from the third day of the menstruation cycle. Follicles were monitored through transvaginal sonography 5–6 days after the gonadotropin stimulation. Cetrotide (0.25 mg/d; Merck Serono, Germany) was prescribed in day 6 after the stimulation cycle until the trigger day. When the diameter of the largest follicle reached 18 mm and the diameter of three follicles exceeded 16 mm, hCG 250 µg (Merk Serono, Germany) was prescribed for the final maturation of oocytes and ovulation.

Oocyte retrieval was performed 36 h after the trigger day. Retrieved cumulus–corona oocyte complexes were washed with equilibrated G-MOPS (Vitrolife) and were then placed into Petri dishes containing G-IVF PLUS (Vitrolife) in a 5% O_2_, 6% CO_2,_ 37 °C incubator (Astec).

### Antral follicle count

Ovarian antral follicles were identified and counted using transvaginal ultrasound (Philips iU22) on menstrual cycle day 2 to 5 for poor ovarian responders with or without Cai’s Prescription treatment.

### Embryo evaluation

After oocyte retrieval, oocytes were fertilized within 5 h by conventional IVF or ICSI, until oocytes were checked for two visible pronuclei to confirm normal fertilization [18]. Then the embryo quality grading were done on day 3 after retrieval as grades 1–6 according to the evenness of each blastomere and the percentage of fragmentation [19]. The embryos comprising 6–8 cells and those of grade 1 or 2 were regarded as high quality embryos [20].

### Library preparation for transcriptome sequencing and data analysis

To reduce the differences between individuals, ovarian granulosa cell samples from five patients were mixed together to extract RNA. A total amount of 1 µg total RNA per sample was used as input material for the RNA sample preparations. Sequencing libraries were generated using NEBNext UltraTM RNA Library Prep Kit for Illumina (New England Biolabs) following manufacturer’s recommendations. The libraries were sequenced on an Illumina Novaseq6000 platform by Shanghai Jiayin Biotechnology.

For bioinformatics analyses, raw sequence reads were initially processed using FastQC for quality control, and then adapter sequences and poor quality reads were removed using Cutadapt. Quality-filtered reads were then mapped to human genome (hg19) using STAR, and only the uniquely mapped reads were kept. Read counts were calculated using HTSeq-count. Differentially expressed genes were identified using R package DESeq2 (fold change ≥ 2, *P* < 0.05 or fold change -2, *P* < 0.05). For Gene ontology (GO) analysis, Fisher’s exact test was applied to identify the significant GO categories and FDR was used to correct the *P* values. Pathway analysis was performed in edgeR via Fisher’s exact test using Kyoto Encyclopedia of Genes and Genomes (KEGG) pathway enrichment analysis.

### Plasmid construction

The CDS region of *ARHGAP4* was amplified and cloned into pCMV-tag 3 A plasmid (Thermo Fisher Scientific) at the BamHI and HindIII sites to obtain pCMV-tag 3 A-ARHGAP4 plasmid.

### Cell culture and transfection

Human ovarian granulosa-like tumor cell line KGN was maintained in DMEM (Corning) with 10% FBS (Biological Industries), 100 U/ml penicillin and 0.1 mg/ml streptomycin sulfate (Gibco) at 37˚C with 5% CO_2_. For transfection, KGN cells were transfected with the indicated *ARHGAP4* siRNA (si*ARHGAP4*: GCCAAGTTCATGGAGCACAAA, RiboBio) or pCMV-tag 3A-ARHGAP4 plasmid using Lipofectamine 3000 transfection reagent (Thermo Fisher Scientific) according to the manufacturer′s instructions. Briefly, KGN cells seeded in 12-well plate with 70-80% confluence were transfected with Lipofectamine 3000 and 20 nM siRNAs or 0.5 µg plasmids. After transfection for 48 h, the culture supernatant was collected for ELISA detection, and cells were harvested for qPCR, immunoblotting or annexin-V and propidium iodide (PI) staining.

### Quantitative real-time PCR (qPCR)

Total RNAs were extracted and purified from granulosa cells from poor ovarian responders or KGN cells using Trizol reagent (Invitrogen). The purified RNAs were dissolved in RNase-free H_2_O. 1 µl of RNA was diluted and subjected to spectrophotometer to detect absorbance at 260 nm and 280 nm. RNA concentration and purity were calculated and evaluated basing on the absorbance value. 2 µg total RNA was reversely transcribed by using M-MLV Reverse Transcriptase (Promega) with random hexamer primers to synthesize single-stranded complementary DNA (cDNA). The cDNA product was then amplified by using FastStart Universal SYBR Green Master (Roche) on Applied Biosystems 7900 Real-Time PCR Systems. The qPCR primers were (forward and reverse, respectively) 5’- TGGTGGAGAGCTGCATTCGCTT-3’ and 5’- CTCGAAGGCATCACGGATCTCT-3’ for *ARHGAP4*; 5’- CCTGTTGTGCTCTTCCATCCTG-3’ and 5’-GGGTTGTAGTTGTGTCACTGG-3’ for *ARHGAP30*; 5’- GGAGGTCAGCAAGGAACGG-3’ and 5’- CAGAGTGGAAGCTAGACGCATG-3’ for *ARHGAP45*; 5’- CACCATTGGCAATGAGCGGTTC-3’ and 5’- AGGTCTTTGCGGATGTCCACGT-3’ for *Actin*. The relative expression of targeted gene was normalized to *Actin* and represented the fold change in expression (2^−△△Ct^).

### Cell proliferation assay (CCK-8)

A total of 5 × 10^3^ KGN cells/well were added into a 96-well plate. After treatment, the proliferative activity was determined at the end of indicated experimental periods (0, 24, 48, 72 and 96 h) using CCK-8 assay kit (Beyotime Biotechnology) according to the manufacturer’s instructions. The absorbance value of each well at a wavelength of 450 nm was determined on a microplate reader (Thermo Fisher Scientific).

### Apoptosis analysis

1 × 10^5^ cells/well were seed into 6-well-plate and cultured overnight for adhesion. After treatment, cells were harvested and stained with annexin-V and PI staining kit (Beyotime Biotechnology) according to the manufacturer’s instructions. The detection of apoptotic cells was performed and analyzed by a flow cytometer (BD FACSAria III).

### Western blot

Total proteins were extracted and purified from granulosa cells from poor ovarian responders or KGN cells using RIPA reagent (Beyotime Biotechnology). The concentration of purified proteins was analyzed by using BCA Protein Assay Kit (Thermo Fisher Scientific). For western blot, 20 µg of protein was subjected to SDS-polyacrylamide gel, followed by transferring to PVDF membrane (Millipore). The membrane was then subjected to blocking with 5% BSA, then incubation with primary antibodies against ARHGAP4 (1:1000), Bax (1:2000) from Abcam, AKT (1:2000), pAKT (Ser473) (1:1000), S6 Ribosomal Protein (1:2000), Phospho-S6 Ribosomal Protein (Ser240/244) (1:1000), Cleaved Caspase-3 (1:1000) from Cell Signaling Technology and Actin (1:10000) from Sigma-Aldrich, and finally incubation with AffiniPure-conjugated corresponding secondary antibody (Sigma-Aldrich) (1:1000–5000). The targeted protein in membrane was visualized using an enhanced ECL kit (Thermo Fisher Scientific). The expression level of Actin was considered as control.

### ELISA

Fifty-thousand cells were seeded in 2 mL media in triplicate wells in 6-well plates (~5,000 cells/cm2). The following day (Day 1), media was replaced with fresh media containing 2 µM cisplatin. Media was replaced on days 3 and 5. On day 6, 1000 µL media was centrifuged at 2000 g. for 5 min at 4° C to pellet cells. 200 µL of media was transferred to round-bottom 96-well plates. ELISAs were performed according to manufacturer instructions.

Serum for FSH and AMH analysis was collected on menstrual cycle day 2 or 3 from poor ovarian responders with or without Cai’s Prescription treatment. Serum for E_2_ detection was collected when 3 leading follicles reached ≥ 18 mm in diameter. The culture supernatant of KGN cells with indicated treatment were collected. ELISA kit of IL-1β, INFγ, FSH, E_2_ and AMH were obtained from JingMei Biotechnology and detections were performed according to the manufacturer’s instructions.

### Statistical analysis

In this study, GraphPad Prism software was used for statistical analysis. Measurement data were statistically described by median. Statistical significance was assessed by two-tailed unpaired Student’s t-test or Mann-Whitey U-test. Differences were considered statistically significant at *P* < 0.05.

## Results

### Transcriptome differences in normal and poor ovarian responders

**Fig. 1:**
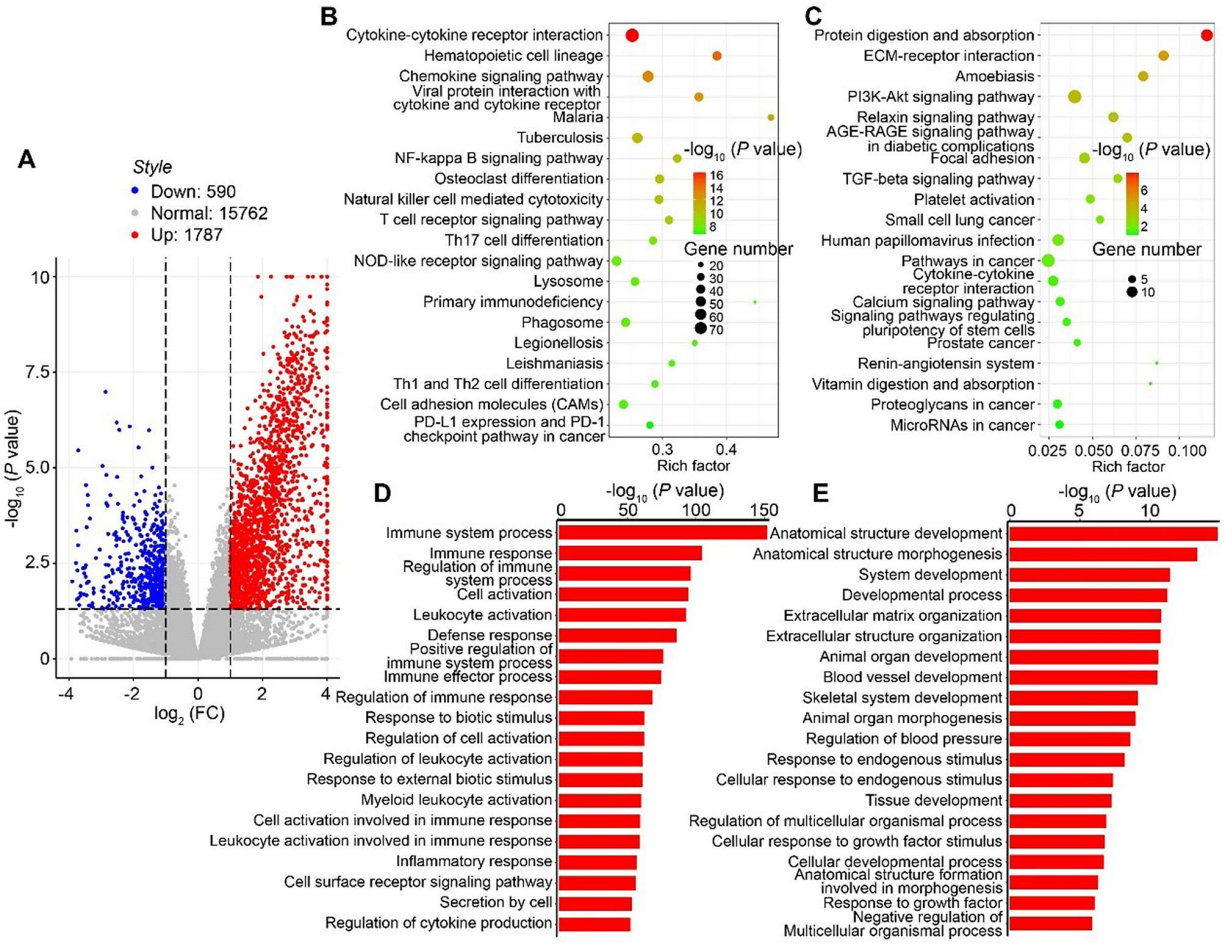
Analysis of gene expression data in normal and poor ovarian responders. (**A**) Volcano plot comparing the differentially expressed genes detected by high-throughput sequencing in granulosa cells between normal and poor ovarian responders. (**B**-**C**) KEGG enrichment analyses of the differentially expressed genes in granulosa cells between normal and poor ovarian responders. (**B**) The top 20 upregulated KEGG pathways. (**C**) The top 20 downregulated KEGG pathways. (**D**-**E**) Gene Ontology (GO) functional enrichment analyses of the differentially expressed genes in granulosa cells between normal and poor ovarian responders. (**D**) The top 20 upregulated GO enrichment scores. (**E**) The top 20 downregulated GO enrichment scores

Ovarian granulosa cells are known to progressively proliferate and differentiate to promote ovulation and undergo luteinization during follicular development [21]. To investigate the underlying mechanism of POR, the mRNA expression levels of ovarian granulosa cells in poor ovarian responders were detected with next generation sequencing. We identified 2377 genes that were differentially expressed (*P* < 0.05), with 1787 up-regulated in the POR (Fig. 1A). KEGG enrichment analyses of these DEGs (differentially expressed genes) were performed to determine the metabolic processes and signal transduction pathways. The results revealed the up-regulated genes are significantly enriched in “Cytokine-cytokine receptor interaction”, “Chemokine signaling pathway”, “NF-κB signaling pathway”, etc. (Fig. 1B). The down-regulated genes are significantly enriched in “Protein digestion and absorption”, “ECM-receptor interaction”, “PI3K-Akt signaling pathway”, “AGE-RAGE signaling pathway”, etc. (Fig. 1C). In addition, Gene Ontology (GO) functional enrichment analyses of the DEGs in granulosa cells also showed that immune related pathways, such as “immune system process”, “immune response”, “regulation of immune system process” were up-regulated (Fig. 1D, E). These data implied the immune response were significantly increased in poor ovarian responders.

### The effects of Cai’s prescription for poor ovarian responders

**Fig. 2:**
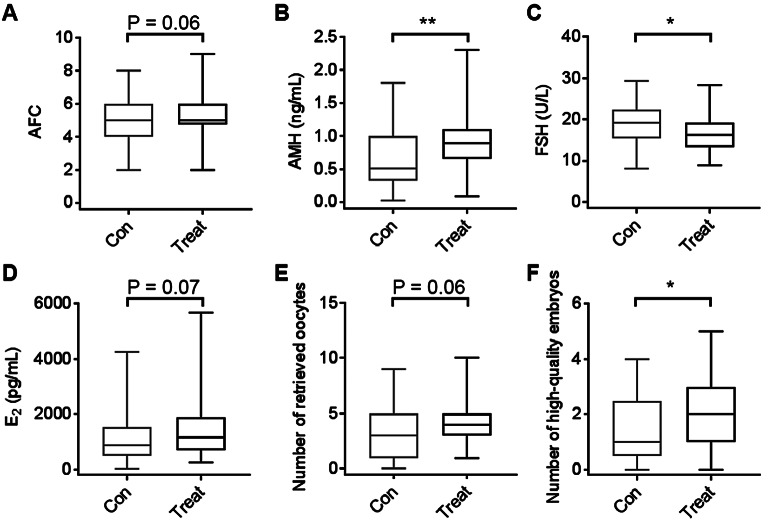
The effects of Cai’s Prescription for poor ovarian responders. (**A**) The antral follicle count (AFC) of poor ovarian responders with or without Cai’s Prescription treatment. (**B**-**D**) Anti-Mullerian hormone (AMH), follicle-stimulating hormone (FSH) and E_2_ levels were quantified via ELISA from serum in poor ovarian responders with or without Cai’s Prescription treatment. (**E**-**F**) The number of retrieved oocytes and high-quality embryos obtained from poor ovarian responders with or without Cai’s Prescription treatment. Boxes represent median and interquartile range; whiskers represent minimum and maximum

To evaluate the effect of Cai’s Prescription for POR in ART, poor ovarian responders were treated with Cai’s Prescription for three months. Compared with untreated poor ovarian responders, AFC was increased (*P* = 0.06, Fig. 2A). The level of AMH increased dramatically after treated with Cai’s Prescription (Fig. 2B), whereas follicle-stimulating hormone (FSH) was significantly decreased (Fig. 2C). In addition, the level of E_2_ also increased after treated with Cai’s Prescription (*P* = 0.07, Fig. 2D). Particularly, the number of retrieved oocytes was increased (*P* = 0.06, Fig. 2E) and the number of high-quality embryos significantly increased (Fig. 2F) after ovarian stimulation with Cai’s Prescription pretreatment. These data showed that pretreatment with Cai’s Prescription in an IVF cycle had the tendency to improve the ovarian reserve function and could enhance the number of high-quality embryos for poor ovarian responders.

### Transcriptome differences in poor ovarian responders with Cai’s prescription

**Fig. 3:**
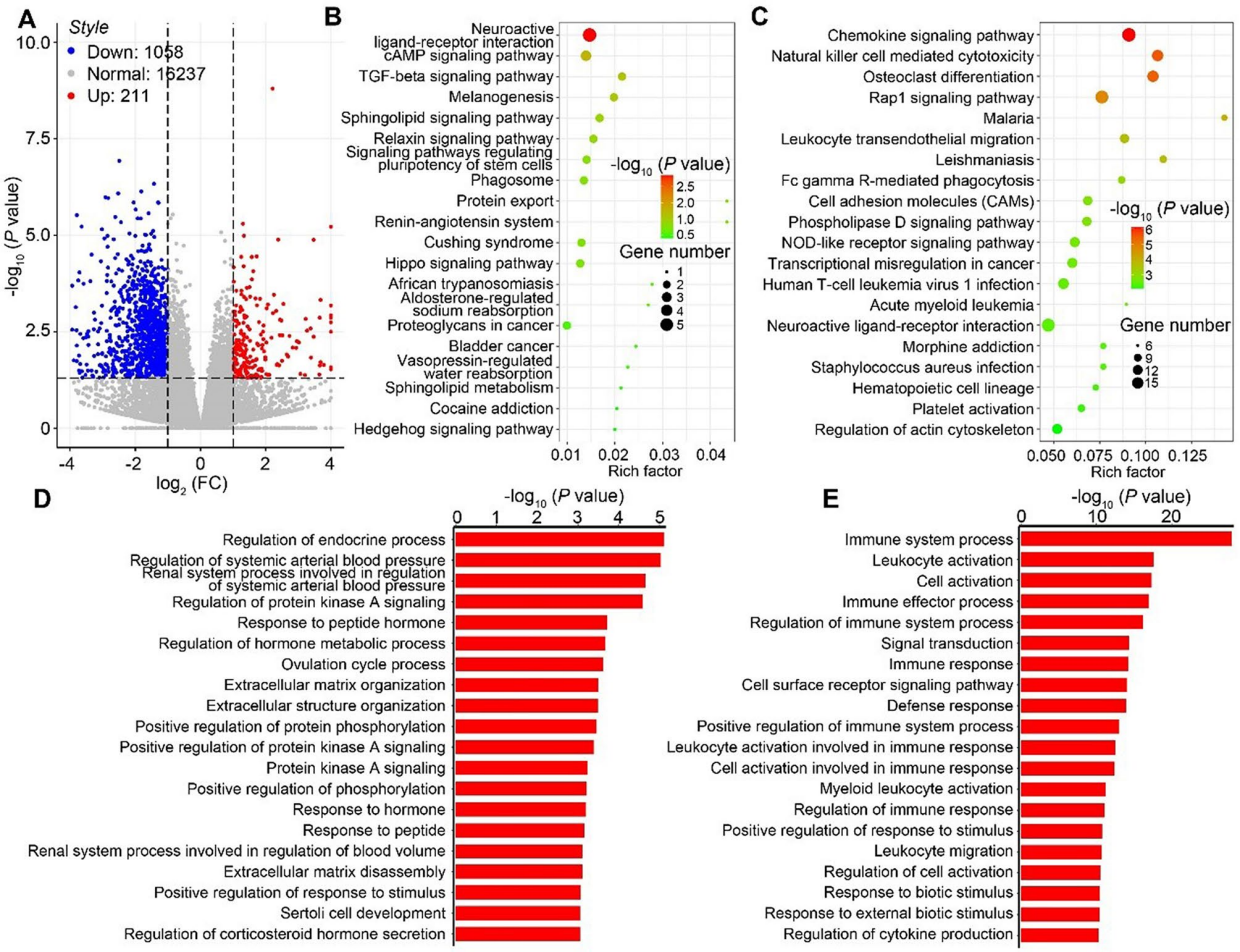
Analysis of gene expression data in poor ovarian responders with or without Cai’s Prescription treatment. (**A**) Volcano plot comparing the differentially expressed genes detected by high-throughput sequencing in granulosa cells from poor ovarian responders with or without Cai’s Prescription treatment. (**B**-**C**) KEGG enrichment analyses of the differentially expressed genes in granulosa cells from poor ovarian responders with or without treatment with Cai’s Prescription. (**B**) The top 20 upregulated KEGG pathways. (**C**) The top 20 downregulated KEGG pathways. (**D**-**E**) Gene Ontology (GO) functional enrichment analyses of the differentially expressed genes in granulosa cells from poor ovarian responders with or without Cai’s Prescription treatment. (**D**) The top 20 upregulated GO enrichment scores. (**E**) The top 20 downregulated GO enrichment scores

To explore the effect of Cai’s Prescription mechanically, the gene transcription patterns of ovarian granulosa cells in poor ovarian responders with or without Cai’s Prescription pretreatment were analyzed. We identified 1269 genes that were differentially expressed (*P* < 0.05), with 1058 down-regulated after the Cai’s Prescription treatment (Fig. 3A). KEGG analysis showed that the up-regulated genes are significantly enriched “Neuroactive ligand-receptor interaction” and “cAMP signaling pathway” (Fig. 3B) and down-regulated genes are significantly enriched in “Chemoskine signaling pathway”, “Natural killer cell mediated cytotoxicity” (Fig. 3C). Moreover, “Regulation of endocrine process”, “Response to peptide hormone”, “Regulation of hormone metabolic process”, “Ovulation cycle process”, “Response to hormone” appeared in the top 20 up-regulated enriched processes (Fig. 3D). The top 5 downregulated GO enrichment processes are “immune system process”, “cell activation”, “leukocyte activation”, “immune effector process”, “regulation of immune system process” (Fig. 3E), with most of them focus on inflammation processes. These data indicated Cai’s Prescription can inhibit immune process to improve ovarian hyporesponsiveness.

### Cai’s prescription downregulates *ARHGAP4* in POR

**Fig. 4:**
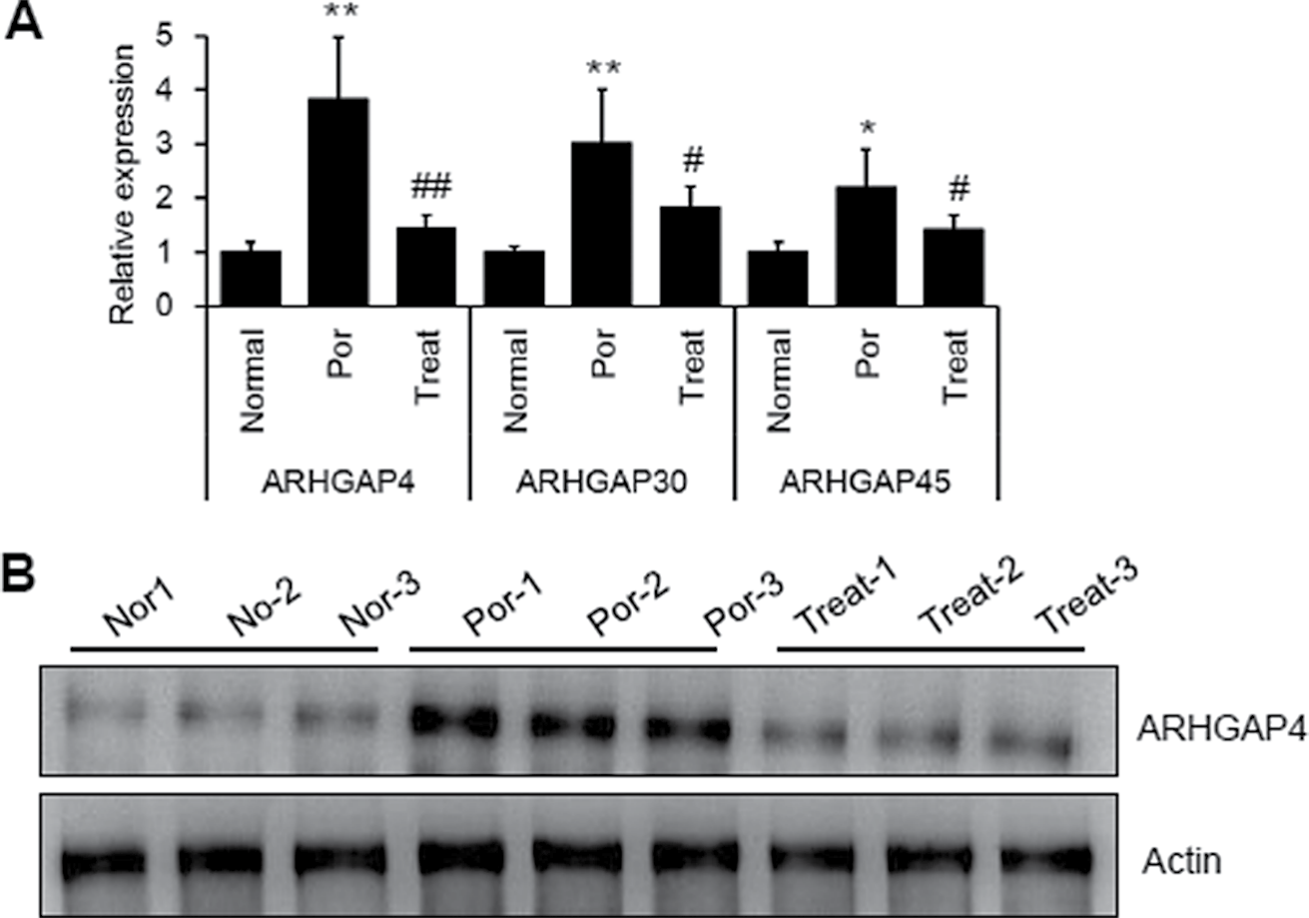
Cai’s Prescription downregulates ARHGAP4 level in granulosa cells from poor ovarian responders. (**A**) The mRNA levels of 3 members from ARHGAP family in granulosa cells from normal, poor ovarian responders and poor ovarian responders after Cai’s Prescription treatment. Data are mean ± SD for three independent experiments. (**B**) The protein level of ARHGAP4 in granulosa cells as described in **A**

To investigate the biological mechanism for Cai’s Prescription treatment, we screened the transcriptome sequencing data with the following criterion: (1) The gene expression level was differentially changed in POR (Por vs. Nor, Log_2_ FC > 1.5); (2) The gene expression level can be rescued after Cai’s Prescription treatment (Treat vs. Por, Log_2_ FC < -1.5). Interestingly, we found three members of GTPase-activating proteins: *ARHGAP4*, *ARHGAP30* and *ARHGAP45*, matched the criterion (Supplementary Table 1). The three candidate genes were confirmed by quantitative PCR, and *ARHGAP4* showed the most intense change (Fig. 4A). The protein level of ARHGAP4 was further analyzed by western blot (Fig. 4B). We chose *ARHGAP4* as the candidate in the following study.

### *ARHGAP4* regulates cell proliferation and inflammation in vitro

**Fig. 5:**
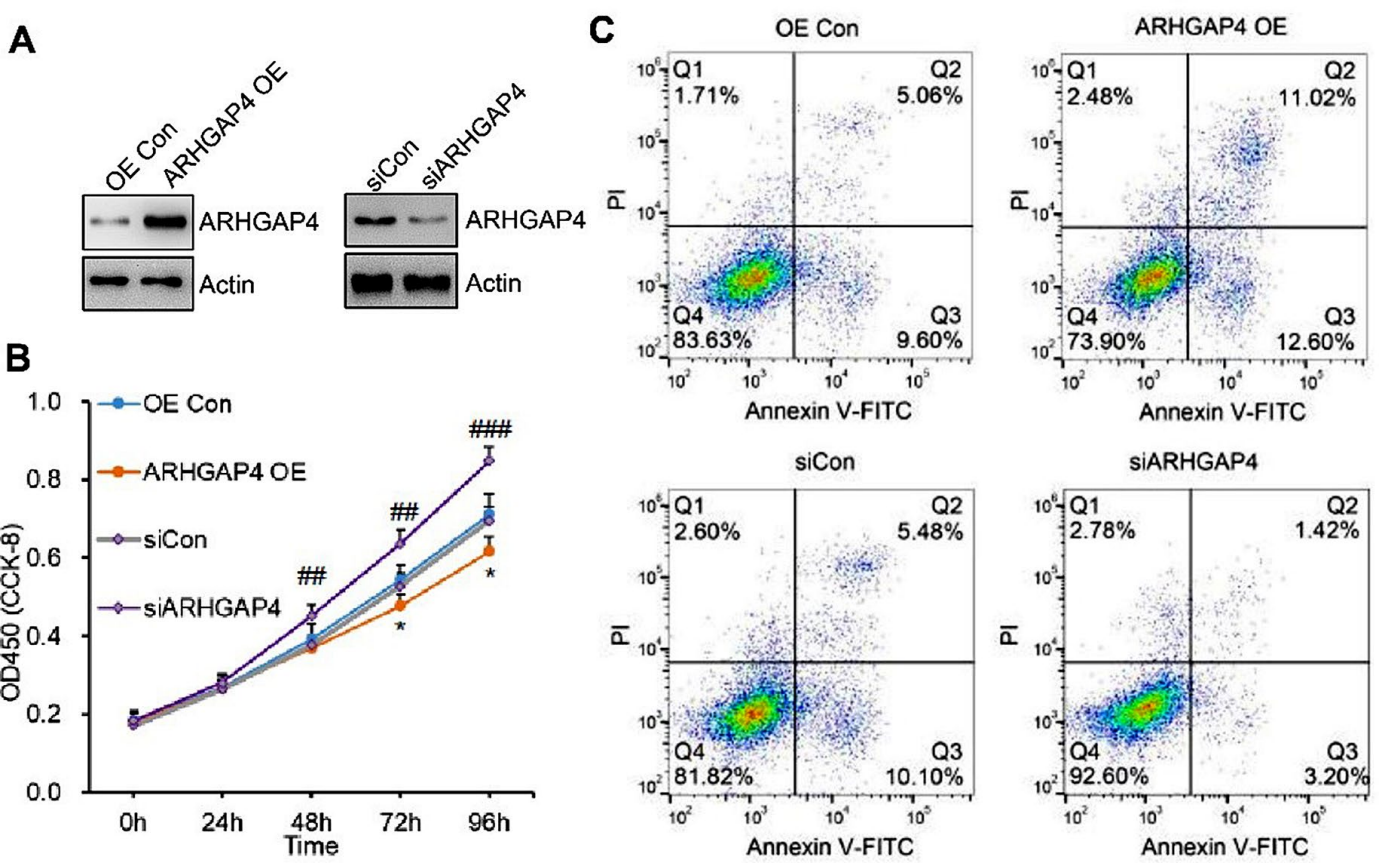
ARHGAP4 regulates cell proliferation and apoptosis in vitro. (**A**) The expression level of ARHGAP4 was detected by western blot in KGN cells transfected with pCMV-tag 3A-ARHGAP4 or ARHGAP4 siRNA. (**B**) CCK-8 assay showed that ARHGAP4 overexpression significantly decreased the ability of KGN cell proliferation, and downregulation of ARHGAP4 showed inverse effect. Data are mean ± SD for three independent experiments. (**C**) Flow cytometry analysis of annexin-V and propidium iodide (PI) staining of apoptotic KGN cells with ARHGAP4 overexpression or downregulation

To examine the function of *ARHGAP4* in granulosa cells, KGN cells were transfected with pCMV-tag 3 A-*ARHGAP4* plasmid or *ARHGAP4* siRNA to overexpress or knockdown of *ARHGAP4* protein level (Fig. 5A), respectively. As shown in Fig. 5B, overexpression of *ARHGAP4* significantly decreased granulosa cell proliferation, whereas down regulation of *ARHGAP4* with siRNA enhanced cell proliferation. Furthermore, cell apoptosis was significantly induced after *ARHGAP4* overexpression, and *ARHGAP4* siRNA treatment showed an inverse effect (Fig. 5C).

**Fig. 6:**
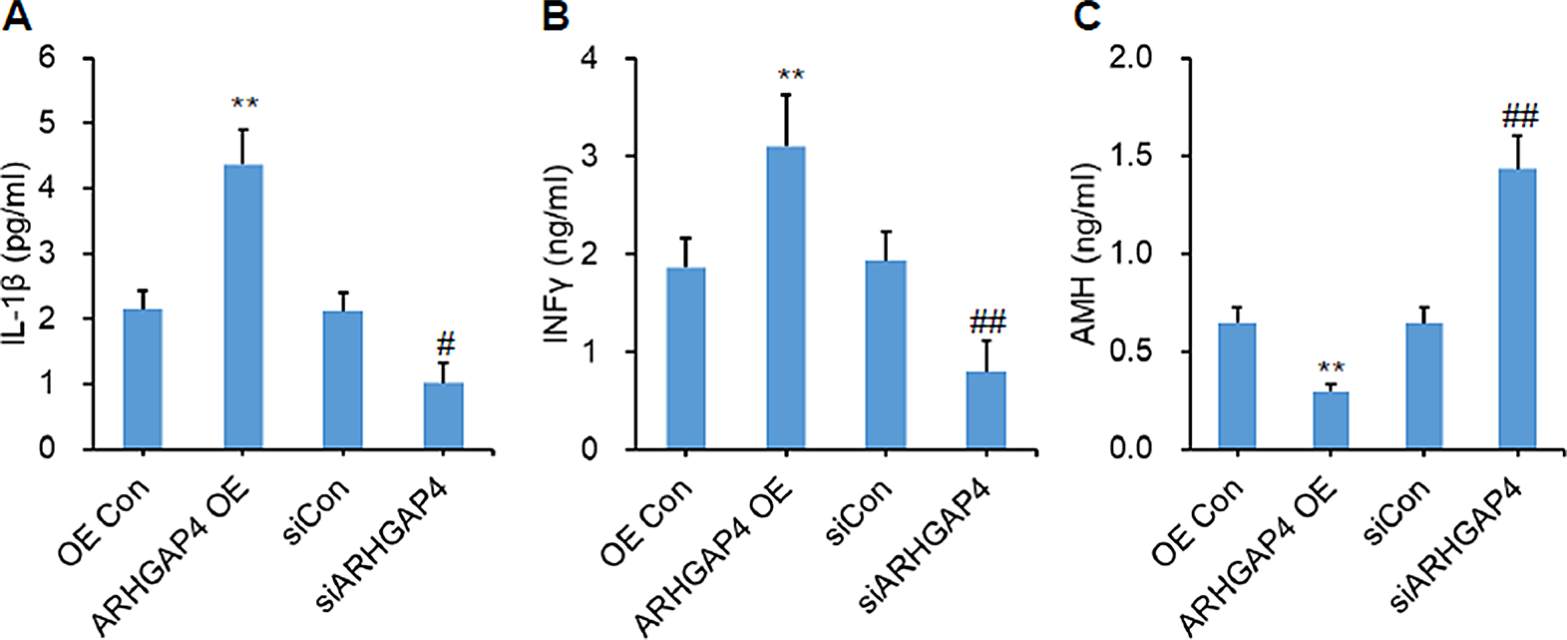
ARHGAP4 regulates inflammation and AMH level. IL-1β (**A**), INFγ (**B**) and AMH (**C**) levels were quantified via ELISA from the supernatant of KGN cells with indicated treatments. Data are mean ± SD for three independent experiments

The inflammation response increased significantly in poor ovarian responders and can be rescued after Cai’s Prescription pretreatment, then we measured the levels of IL-1β, INFγ in KGN cell medium. Interestingly, both IL-1β and INFγ levels were significantly upregulated with *ARHGAP4* overexpression. On the contrary, IL-1β and INFγ levels can be inhibited when *ARHGAP4* was knocked down (Fig. 6A, B). Moreover, AMH level also showed negative correlation with *ARHGAP4* expression (Fig. 6C). These data indicated *ARHGAP4* can inhibit granulosa cell proliferation and promote inflammation in vitro.

### Cai’s prescription regulates cell apoptosis via PI3K-Akt pathway

**Fig. 7:**
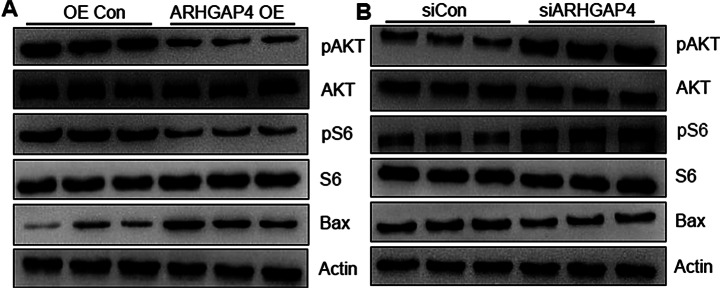
ARHGAP4 regulates cell apoptosis through PI3K-Akt pathway. (**A**) The protein level of Bax was increased, while phosphorylation levels of AKT, S6 were decreased in KGN cells with ARHGAP4 overexpression. (**B**) The protein level of Bax was decreased, while phosphorylation levels of AKT, S6 were increased in KGN cells with *ARHGAP4* downregulation

To investigate the role of *ARHGAP4* mechanically, we focused on PI3K-Akt signaling pathway, which was downregulated in poor ovarian responders. As shown in Fig. 7A, the protein level of pAKT and pS6 were significantly decreased with ARHGAP4 overexpression. However, Bax, a key apoptosis regulator suppressed by activation of PI3K-Akt signaling pathway, increased dramatically when ARHGAP4 overexpression in KGN cells. Conversely, PI3K-Akt signaling pathway was activated when *ARHGAP4* expression was knocked down (Fig. 7B).

**Fig. 8:**
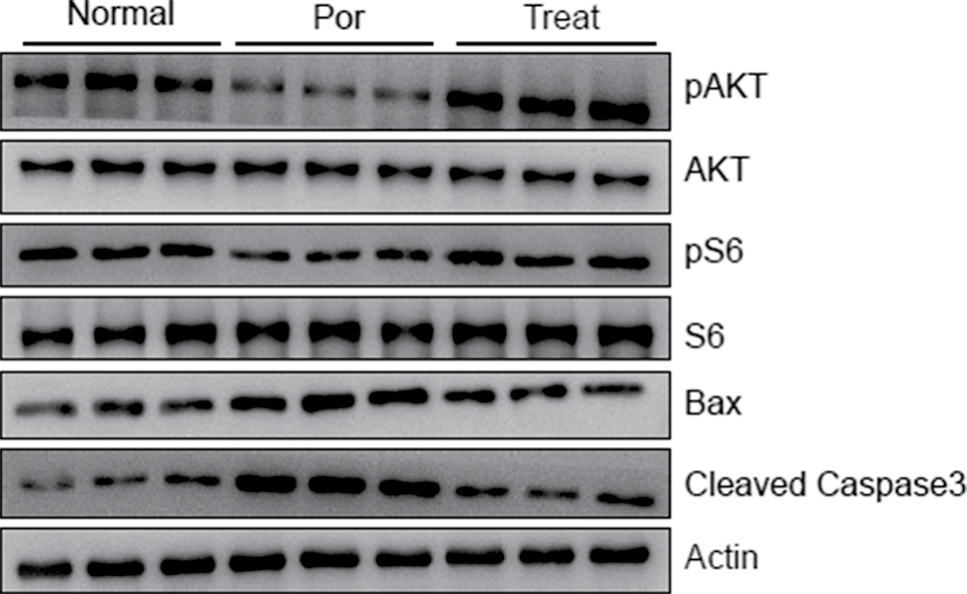
Cai’s Prescription regulates PI3K-Akt pathway. The protein levels of Bax, Cleaved Caspase3, AKT, S6, and phosphorylation of AKT, S6 were analyzed in granulosa cells from normal, POR and POR after Cai’s Prescription treatment patients

Moreover, PI3K-Akt signaling pathway was inhibited in poor ovarian responders, and could be rescued by Cai’s Prescription pretreatment partially (Fig. 8). In addition, apoptosis was inhibited after Cai’s Prescription pretreatment as the protein levels of Bax and Cleaved Caspase3 recovered to normal (Fig. 8).

## Discussion

TCM, as a kind of alternative holistic therapies, offer less invasive and less costly physical and emotional treatment compared with standard Western Medical treatment [22]. TCM multi-channel interventional therapy yields satisfactory results in different diseases which has influenced the opinions of people in China and the surrounding areas, particularly for people with infertility. Here we found that inflammation response of ovarian granulosa cells in poor ovarian responders was significantly increased. After Cai’s Prescription pretreatment in poor ovarian responders, inflammation was declined significantly, ovarian reserve function improved, and the number of high quality embryos increased. Mechanically, we discovered Cai’s Prescription pretreatment reduced inflammation response and cell apoptosis in poor ovarian responders, and activated PI3K-Akt signaling pathway through *ARHGAP4*.

TCM therapy for infertility is often not suggested by Western Medical practitioners because of the molecular mechanism of their action needs to be elucidated specifically. However, plenty of studies have reported that management of infertility with TCM can improve pregnancy rates successfully involving inhibition of inflammation [23–25]. It has been reported that pro-inflammatory immune responses may impair the ovarian response [26]. Cai’s Prescription components comprise 11 herbs: Fuling, Shengdihuang, Shudihuang, Nvzhenzi, Xianmao, Xianlingpi, Bajitian, Roucongrong, Lujiaoshuang, Zishiying, and Ziheche. From our clinical study and high throughput sequencing data of ovarian granulosa cells, the inflammation response was diminished extendedly after Cai’s Prescription pretreatment. Inflammatory cytokines can regulate aspects of follicular development, such as growth, differentiation and death [27]. The Chinese herbs Fuling, Shengdihuang, Nvzhenzi, etc., containing antioxidants and flavonoids, have exerted anti-inflammatory activity in different cells [28–30]. These observations imply that Cai’s Prescription can be an effective compound medicines to diminish inflammation response because of some compounds in the prescription.

GTPase-activating protein (GAP), a negative regulator of GTPase protein, is thought to catalyze the conversion of the active GTPase-GTP form to the inactivate GTPase-GDP form. It has been reported that Rho family GTPases function as “molecular switch” in cellular signaling regulation processes including inflammation and apoptosis [31]. For example, there is a positive correlation between RhoA and TNF-α in the intestinal inflammatory tissues of Crohn’s disease patients, and RhoA/ROCK pathway activation stimulates the production of TNF-α and IL-1β [32]. Decreased RhoA activation has been reported to contribute to T-cell apoptosis with decreased expression of Bcl-xl and pBad [33]. However, the function of Rho family GTPase in granulosa cells is still unclear. Here we found *ARHGAP4*, a member of the RhoGAPs family, involves in the inflammation and apoptosis via PI3K-Akt pathway in granulosa cells. The expression level of *ARHGAP4* was significantly up-regulated in poor ovarian responders, transforming the Rho GTPases to inactive GDP-bound form. Then the PI3K-Akt pathway was suppressed, leading to inflammation and apoptosis. On the contrary, Cai’s Prescription may reverse the inflammation response and cell apoptosis via inhibiting *ARHGAP4*. It has been reported that *ARHGAP4* can be a novel regulator of in pancreatic cancer cells [34]. Moreover, the high expression of *ARHGAP4* in colorectal cancer is related to the immune cells such as B cells, CD8^+^ and CD4^+^ T cells, macrophages, neutrophils, and dendritic cells [35]. *ARHGAP4* maybe a new target for POR treatment.

Taken together, our study provided key evidences that management of female infertility with Cai’s Prescription was beneficial for poor ovarian responders, leading to increase of the number of high quality embryos, and *ARHGAP4* may be a candidate target for POR treatment.

### Electronic supplementary material

Below is the link to the electronic supplementary material.

**Supplementary Table 1**. Transcriptome differences in poor ovarian responders with or without Cai’s Prescription treatment.

## Data Availability

The datasets used and analyzed during the current study are available from the corresponding author on reasonable request.
